# Organogel Coupled with Microstructured Electrospun Polymeric Nonwovens for the Effective Cleaning of Sensitive Surfaces

**DOI:** 10.1021/acsami.0c09543

**Published:** 2020-08-21

**Authors:** Yiming Jia, Giorgia Sciutto, Rocco Mazzeo, Chiara Samorì, Maria Letizia Focarete, Silvia Prati, Chiara Gualandi

**Affiliations:** † Department of Chemistry “G. Ciamician”, Microchemistry and Microscopy Art Diagnostic Laboratory (M2ADL), University of Bologna, Via Guaccimanni 42, 48121 Ravenna, Italy; ‡ Chongqing Cultural Heritage Research Institute, 400013 Chongqing, China; § Department of Chemistry “G. Ciamician”, University of Bologna, Via Sant’Alberto 163, 48123 Ravenna, Italy; ∥ Department of Chemistry “Giacomo Ciamician” and INSTM UdR of Bologna, University of Bologna, Via Selmi 2, 40126 Bologna, Italy; ⊥ Health Sciences & Technologies (HST) CIRI, University of Bologna, Via Tolara di Sopra 41/E, 40064 Ozzano Emilia Bologna, Italy; # Interdepartmental Center for Industrial Research on Advanced Applications in Mechanical Engineering and Materials Technology, CIRI-MAM, University of Bologna, Viale Risorgimento, 2, 40136 Bologna, Italy

**Keywords:** organogel, polyhydroxyalkanoates, electrospinning, composite material, Dammar varnish

## Abstract

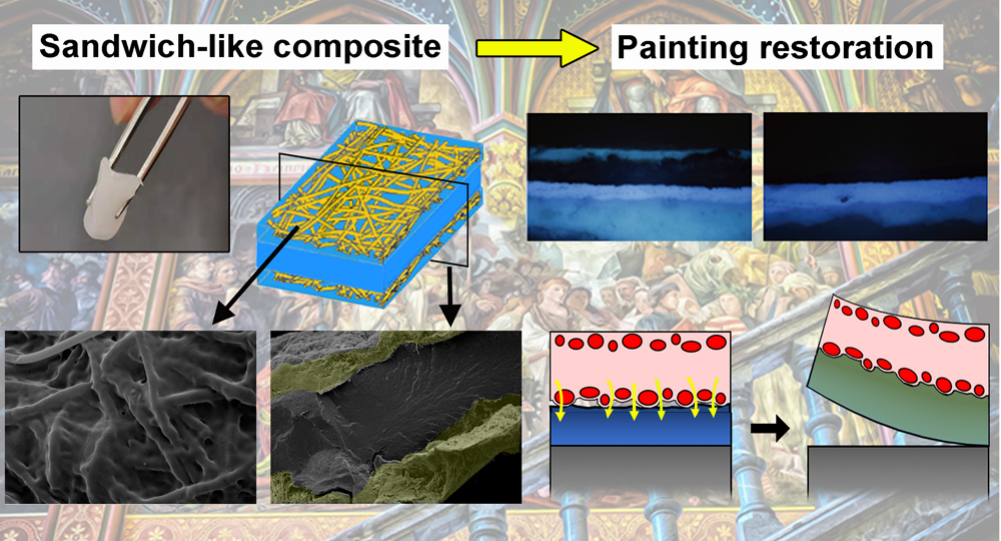

Hydrogels and organogels are widely used as cleaning materials, especially when a controlled solvent release is necessary to prevent substrate damage. This situation is often encountered in the personal care and electronic components fields and represents a challenge in restoration, where the removal of a thin layer of aged varnish from a painting may compromise the integrity of the painting itself. There is an urgent need for new and effective cleaning materials capable of controlling and limiting the use of solvents, achieving at the same time high cleaning efficacy. In this paper, new sandwich-like composites that fully address these requirements are developed by using an organogel (poly(3-hydroxybutyrate) + γ-valerolactone) in the core and two external layers of electrospun nonwovens made of continuous submicrometric fibers produced by electrospinning (either poly(vinyl alcohol) or polyamide 6,6). The new composite materials exhibit an extremely efficient cleaning action that results in the complete elimination of the varnish layer with a minimal amount of solvent adsorbed by the painting layer after the treatment. This demonstrates that the combined materials exert a superficial action that is of utmost importance to safeguard the painting. Moreover, we found that the electrospun nonwoven layers act as mechanically reinforcement components, greatly improving the bending resistance of organogels and their handling. The characterization of these innovative cleaning materials allowed us to propose a mechanism to explain their action: electrospun fibers play the leading role by slowing down the diffusion of the solvent and by conferring to the entire composite a microstructured rough superficial morphology, enabling to achieve outstanding cleaning performance.

## Introduction

Soft hydrogels and organogels combined with suitable solvents are commonly employed for the cleaning of dirty surfaces, especially when the substrate is susceptible to swelling and its functionality is compromised by the solvent itself. This is a frequent issue encountered in art restoration as well as in personal care and electronics. In these frameworks, the benefits of gels rely on their capability to entrap the solvent to minimize substrate damage and to adapt their shape to maximize the contact with the surface, necessary to achieve good cleaning efficiency. A typical application of hydrogels and organogels is the selective removal of varnish layers from paintings.^1−4^ Traditionally, natural resins, such as dammar and shellac, have been widely used for the preparation of varnishes.^5^ With the passing of time, natural resins change their chemical, optical, and mechanical properties because of the oxidative actions of light, air, and the interaction with pollutants.^6,7^ The most common procedure for the removal of the discolored and degraded varnishes consists in applying solvents with cotton swabs. However, this approach is extremely risky for the painting because the solvent action is highly uncontrollable and leads to saponification, swelling, and leaching of the painting layer components, with decrease of the surface mechanical strength.^8−10^ A valid alternative is the use of gels able to exert a controlled solvent action. Consequently, chemical and physical gels have become popular for the removal of aged varnishes or coatings from artwork surfaces through a “press and peel” approach.^1−3,11−13^ Gels used in restoration have different physical aspects: viscous dispersions, sticky masses, flexible, and peelable films.^3,4^ Gel–solvent interaction and chemical/physical cross-links affect both solvent retention and mechanical properties, thus determining the final cleaning efficacy, the presence of gel residues, the safety for the underneath substrate, and the ease of use.^14,15^ Generally, low elastic moduli are desired to maximize the contact with the surface to achieve better cleaning at the expense, however, of retention capability and gel cohesion, with a consequent uncontrolled release of solvent, undesired painting damages, and residues on the object. Therefore, when designing gels for such a kind of applications, the challenge is to find a good compromise between gel softness and solvent retention.

We have recently proposed poly-3-hydroxybutyrate (PHB)-based gels with green solvents as new cleaning systems for paintings and indoor bronzes^16−18^ addressing the demand of green bio-based cleaning approaches,^19−25^ to the benefit not only of the artworks, but also of restorers and the environment. A step forward for optimizing the use of these gels is the improvement of their cleaning efficiency, solvent retention, handling, and mechanical properties in order to avoid their fragmentations when they are applied or removed. To this aim, the combination of gels with mechanical resistant materials in a composite structure is expected to provide relevant benefits.

Nonwoven fabrics of continuous micrometric fibers made by electrospinning technology have unique properties, such as interconnected and open porosity, high surface area, liquid permeability, excellent flexibility, and mechanical resistance,^26,27^ which make them ideal materials to act as reinforcing components for organogels. Electrospinning is a polymer processing technique that uses electrostatic forces to stretch a viscoelastic jet derived from a polymer solution. Electrospun fibers are currently used for a wide range of applications, including filtration, sensors, catalytic systems, energy storage, structural composites, and biomedical applications.^28−31^ Electrospinning is a consolidate technology on a lab-scale dimension and the scaling up toward levels feasible for mass production has been realized in the past decade by several companies.^32^

Electrospun materials have been already used in combination with soft gels for applications in the biomedical field. Here, their unique structures and properties^33^ have been exploited for enhancing gel mechanical properties^34−36^ and various combination strategies have been adopted to obtain different kind of fibers–hydrogel composites.^37−41^ Moreover, the use of fibers with submicrometric diameters confers to the gel a surface microstructuration that is considered highly advantageous for tissue engineering applications.^42^

With these premises, in this work, we propose for the first time the use of new sandwich-like composites based on the combination of electrospun polymers, that is, poly(vinyl alcohol) (PVA) and polyamide 6,6 (PA6,6), with the PHB-based organogel. In these composites, electrospun mats act as external mechanically resistant layers and provide microstructuration on the surface of the composite. The gel constitutes the core and contains the active solvent, which in turn exerts its function by diffusing through the pores of the electrospun layer toward the paint. PA6,6/PHB–GVL and PVA/PHB–GVL composites have been produced and their mechanical properties, morphology, and composition have been characterized. Their capability to remove a varnish layer made with a terpenic material applied on a paint mock-up and prepared by following ancient recipes^43^ was also tested. Finally, a possible mechanism was suggested to explain why the composite materials produce a limited and superficial release of solvent and at the same time are able to exhibit exceptional cleaning performances.

## Experimental Part

### Materials

γ-Valerolactone (GVL), PHB, PVA (*M*_w_ = 85,000–124,000 g/mol, 87–89% hydrolyzed), 1,1,1,3,3,3-hexafluoro-2-propanol (HFIP), absolute ethanol (EtOH), and γ-butyrolactone (BL) were purchased from Sigma-Aldrich. PA6,6 (Zytel E53 NC010) was kindly provided by DuPont. All of the chemical reagents were commercially available and directly used without treatment.

### Fabrication of PVA and PA6,6 Electrospun Nonwoven Fabrics

The electrospinning apparatus, made in-house, was composed by a high voltage power supply (Spellman SL 50 P 10/CE/230), a syringe containing the polymeric solution connected to a stainless steel blunt-ended needle (inner diameter: 0.51 mm), and a grounded aluminum rotating mandrel as a collector (length = 12 cm, diameter = 5 cm). The polymeric solution was dispensed through a polytetrafluoroethylene (PTFE) tube to the needle that was vertically placed on the collector. PVA was dissolved in EtOH/H_2_O (1/1 v/v) at a concentration of 10% w/v and stirred until all the polymer dissolved in the solvent at room temperature (RT). PVA nonwoven was fabricated using the following conditions: applied voltage 16 kV, needle-to-collector distance 18 cm, solution flow rate 1.5 mL/h, at RT, and relative humidity 40–50%. PA6,6 was dissolved in HFIP at a concentration of 20% w/v and stirred until all of the polymer dissolved at RT. PA6,6 nonwoven was fabricated using the following conditions: applied voltage 20 kV, needle-to-collector distance 15 cm, solution flow rate 0.5 mL/h, at RT, and relative humidity 40–50%. Electrospun samples have a final dimension of about 15 × 8 cm^2^ and a thickness in the range 70–110 μm.

### Preparation of the PHB–GVL Organogel

PHB–GVL gels were produced according to a previously reported procedure.^16^ Briefly, 300 mg of PHB were solubilized in 3 mL of GVL, in a closed vial, by stirring at 110 °C for 5 min in an oil bath, until all the PHB powder dissolved in the solution and became transparent. The solution was then poured in a Petri dish and cooled down to RT to obtain an opalescent and stiff gel about 3 mm thick.

### Preparation of the Sandwich-Like Composites

**Figure 1:**
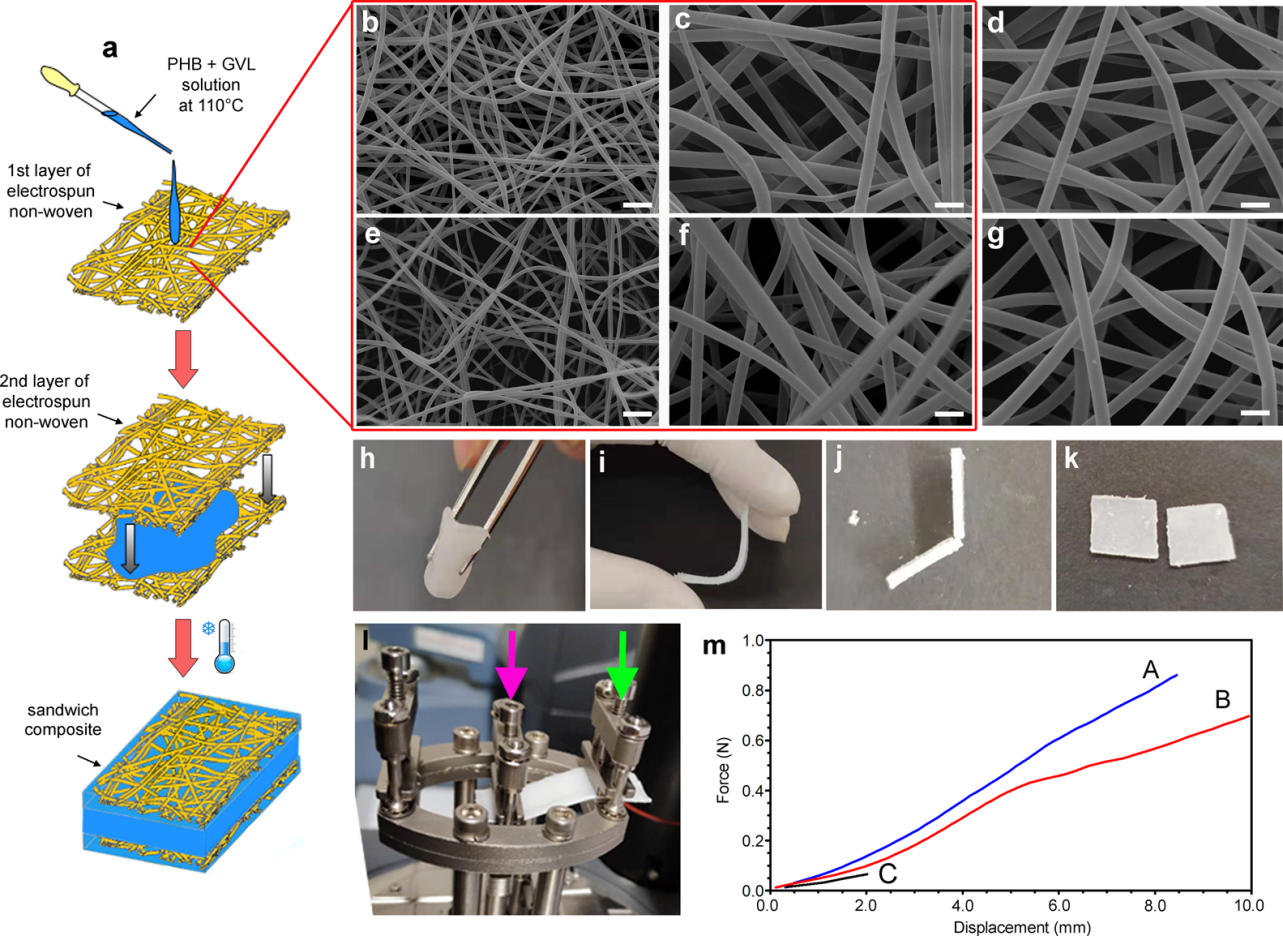
Sketch describing the preparation of PA6,6/PHB–GVL and PVA/PHB–GVL sandwich-like composites (a). SEM images of PA6,6 (b,c) and PVA (e,f) fibers at different magnifications; PA6,6 (d) and PVA (g) fibers after immersion in GVL for 1 min at 110 °C and dried at RT. Representative pictures of sandwich-like composite (h,i) and PHB–GVL gel (j,k) showing the different mechanical resistance of the two types of materials under bending. (l) Single cantilever configuration for sample mounting, where the green arrow indicates the fixed clamp and the pink arrow indicates the movable clamp (a representative composite sample is mounted in the picture). (m) Force-displacement curves of PA6,6/PHB–GVL composite (blue, A), PVA/PHB–GVL composite (red, B), and PHB–GVL gel (black, C): composites resist up to 10 mm bending, whereas the gel breaks at 2 mm. Scale bar: 6 μm (b,e); 2 μm (c,d,f,g).

Figure 1a reports the procedure for the preparation of PVA/PHB–GVL and PA6,6/PHB–GVL sandwich-like composites. PHB–GVL (200 μL) solution prepared at 110 °C was dropped onto a layer of electrospun nonwoven (typically 1 × 1 cm^2^). During this step, the PHB–GVL solution well-impregnated the pores of the electrospun tissue by homogeneously spreading over the entire surface of the nonwoven. A second layer of electrospun nonwoven was quickly laid on the top of the PHB solution by manually applying a slight pressure. The duration of the dropping and of the application of the second layer was controlled to be not longer than 30 s. The solution instantaneously wetted and impregnated the second fabric. The system was finally let to cool down to RT, obtaining a final sandwich-like composite with thickness in the range 1.7–1.9 mm (see Table S1 for details on the geometry of the composites). No shrinkage is observed immediately after the preparation (Figure S1). The composites were employed for mock-ups cleaning within 30 min from the preparation. In this time interval, no relevant modification of the sample shape was detected (Figure S1).

### Characterisation of the PHB–GVL Gel and Sandwich-Like Composites

Sample morphology was observed with a scanning electron microscope (SEM, Leica Cambridge Stereoscan 360) at an accelerating voltage of 20 kV. Prior to SEM analysis, the samples were dried in an oven at *T* = 40 °C for 15 days to remove the GVL solvent, and sputtered with gold. The distribution of fiber diameters was determined through the measurement of about 100 fibers and the results were given as the average diameter ± standard deviation. The student unpaired *t*-test was used to test the statistical significance of the difference between the mean values (*p* < 0.05). Thermogravimetric analyses (TGA) were carried out using a TGA Q500 thermogravimetric analyzer (TA Instruments). The thermograms were recorded from RT to 700 °C at a heating rate of 10 °C/min under the N_2_ atmosphere. TGA analysis was performed twice on the composites to verify the reproducibility of the result. The mechanical characterization of the materials was carried out using a Dynamic Mechanical Analyzer DMA Q800 (TA Instruments) in single cantilever configuration at RT. The analyses were performed on rectangular strips (width 15 mm; gauge length about 18 mm) whose thickness was measured by a digital caliper. Two replicas for each sample were carried out by applying a ramp force of 1 N/min until sample break. Differential scanning calorimetry (DSC) was carried out under the N_2_ atmosphere by means of a differential scanning calorimeter (DSC Q100; TA Instruments) equipped with a refrigerated cooling system. Electrospun samples were kept at 50 °C for 1 h under N_2_ flow (50 mL/min) to eliminate most of the absorbed water and were subsequently subjected to two heating scans at 20 °C/min and one fast cooling applied between the heating scans.

Rheological experiments were carried out on an Anton Paar rheometer MCR 102 using a cone-plate configuration (50 mm diameter). The temperature was controlled by an integrated Peltier system and a Julabo AWC100 cooling system. To keep the sample wet during the measure, a solvent trap was used (H-PTD200). The temperature sweep analyses during cooling rate scans (1, 5 and 10 °C/min) were performed with a fixed gap value of 0.102 mm at a constant strain of 0.5% and frequency of 10 rad/s. The PHB–GVL solutions were prepared at 120 °C and transferred on the hot plate of the rheometer kept at 140 °C. The solution was maintained for 3 min in the hot cone-plate before starting the test. The viscosity of the solution was also monitored during a cooling scan at 10 °C/min by applying a strain rate of 10 1/s.

### Cleaning Procedures

To evaluate the cleaning efficiency, the sandwich-like composites were tested on mock-ups and compared with the PHB–GVL gel. The mockups were prepared according to traditional painting techniques, and naturally aged over 2.5 years. The preparation layer was made of gypsum and rabbit glue, dissolving 1.0 g of animal glue in 5.0 mL of hot distilled water and mixing it with 6.0 g of grinded gypsum. The pigment layer was obtained by dissolving 2.0 g of red burnt ochre in 2.0 mL of 10% w/w of rabbit glue solution. The varnish layer was prepared by dissolving 1.0 g of dammar in 2.5 mL of turpentine. PVA/PHB–GVL, PA6,6/PHB–GVL, and PHB–GVL gel were directly applied on the surface of painting mock-ups for 5 min, then the samples were removed and the surface was further cleaned with dry cotton swabs to eliminate the residues of dammar and the excess solvent.

### Evaluation of the Cleaning Performances

Cross sections of mock-ups varnished with dammar before and after the cleaning treatment were observed by optical microscopy in visible and ultraviolet light (Olympus Optical Microscope BX51, Tokyo, Japan). Attenuated total reflectance (ATR) analyses were performed with a Thermo Nicolet (Thermo Fisher Scientific, Waltham, MA, USA), iN10MX imaging microscope, fitted with a mercury–cadmium–telluride detector cooled by liquid nitrogen. Measurements were performed using a slide-on ATR objective, equipped with a conical germanium crystal, in the range 4000–675 cm^–1^, at a spectral resolution of 4 cm^–1^ with 128 scans and an optical aperture of 150 × 150 μm. Spectroscopic analysis was performed on 3 different areas treated with the same cleaning procedure and 4 spectra were recorded before and after treatment. Headspace solid phase microextraction (HS-SPME) was employed for the evaluation of the solvent retention into the substrate after the cleaning treatments.^16,17^ Analyses were performed exposing directly a carboxen–polydimethylsiloxane fiber into the headspace of a sealed vial containing the sample. The sample consisted in a fragment of around 1.0 mg collected from the surface of the painting mock-up. Because of the low volatility of GVL, sampling was performed after 2 h and after 24 h from the application to better describe the release behavior of the cleaning systems. The sample was placed into a 20 mL HS vial, spiked with 1 μg of the internal standard (BL) and sealed with a silicone/PTFE septa and aluminum cap (Thermo Fisher Scientific, Waltham, MA, U.S.A.). The SPME fiber was inserted into the headspace vial and the sample was thermally heated to 150 °C for 30 min. After reaching the extraction time, the fiber was inserted into the injector of a 5977 Agilent gas chromatograph connected to a 7820A Agilent quadrupole mass spectrometer (Agilent Technologies, Inc., Santa Clara, CA, U.S.A.). Analytes were thermally desorbed at 250 °C for 15 min and separated with a DB-FFAP polar column (30 m length, 0.25 mm i.d., 0.25 μm film thickness), using helium as carrier gas. The thermal program was: 100–250 °C at 10 °C min^–1^. The abundances of the individual compounds were quantified from the *m*/*z* 86 for BL, 100 for GVL mass chromatograms. Calibration curves were performed for BL in the consistent concentration of 1000 ppm in methanol and GVL in the concentration range of 500–8000 ppm by the regression method. The areas corresponding to this range of concentration of standard solutions are in accordance with those obtained by sample measurements, at least two sample replicas treated with the same procedure were performed to quantify the amount of GVL in the substrate after the cleaning procedure.

## Results and Discussion

### Characterisation of the PHB Gel and Sandwich-Like Composites

Nonwoven fabrics made of randomly oriented fibers of PA6,6 and PVA were fabricated by applying optimized electrospinning conditions to gain continuous, smooth, and bead-free fibers (Figure 1b,c,e,f) with comparable submicrometric diameters (790 ± 180 μm for PA6,6 and 750 ± 150 μm, *p* > 0.05). Both electrospun samples are highly flexible and mechanically resistant to handling. PVA is a water-soluble polymer largely employed for pharmaceutical uses, protective water-soluble films, textile processing, and cosmetics. PA6,6 is used for textiles, in automotive, and for electronic goods. Both polymers are semicrystalline (see DSC in Figure S2), strong but ductile, and both are neither solubilized nor swollen by the GVL solvent.

To optimize the procedure for the preparation of the sandwich-like composites, the rheological properties of the PHB–GVL solution were investigated with the aim to identify the temperature and the dropping conditions for obtaining reproducible results. Figure S3 shows that the temperature of gelation of PHB–GVL solution is in the range 75–113 °C, depending on the cooling rate. The data demonstrate that the PHB–GVL system massively changes its property in a narrow temperature range, thus making the preparation of the composite critical if not performed in the proper conditions. In particular, it was experimentally verified that, to achieve the formation of composites with cohesive layers, the dropping step and the laying of the second electrospun mat must be performed in a time range of about 30 s, before the occurrence of PHB–GVL gelation. It was also verified that, as soon as the PHB–GVL is in the liquid state, the viscosity is not affected by the temperature (Figure S3d), meaning that its percolation in the pores of the electrospun layers is not expected to be influenced by the solution temperature. This information allowed to set the proper dropping conditions in terms of temperature and duration of the procedure, as reported in the “Experimental Part” section.

The stability of the fibers at the PHB–GVL solution was evaluated by means of SEM and DSC. Figure 1d,g shows that the fiber morphology is perfectly preserved in contact with GVL at 110 °C (i.e., at the temperature of composite preparation). DSC analyses demonstrate that the solid-state structure of fibers, in terms of the amorphous-to-crystalline ratio, is also preserved after GVL treatment (see Figure S2 and the Supporting Information for details).

The sandwich-like composites appear highly flexible and resist to handling and bending (Figure 1h,i). A remarkable different behavior is displayed by the PHB–GVL gel, that presents a fragile behavior and breaks when slightly bended (Figure 1j,k). The mechanical resistance of the different materials was better evaluated by using a DMA analyzer in single cantilever configuration (Figure 1l). Here, the sample was mounted on one side to the fixed clamp (green arrow) and on the other side to the movable clamp (pink arrow). Force-displacement curves are shown in Figure 1m and clearly show that sandwich-like composites can be bended up to 1 cm of lateral displacement before breaking and resist to higher forces compared to PHB–GVL gels, which instead breaks after a minimal bending. Therefore, in the sandwich composites, the excellent resistance to handling and deformation of the external layers of electrospun nonwovens is successfully transferred to the gel, accomplishing improved mechanical properties.

**Figure 2:**
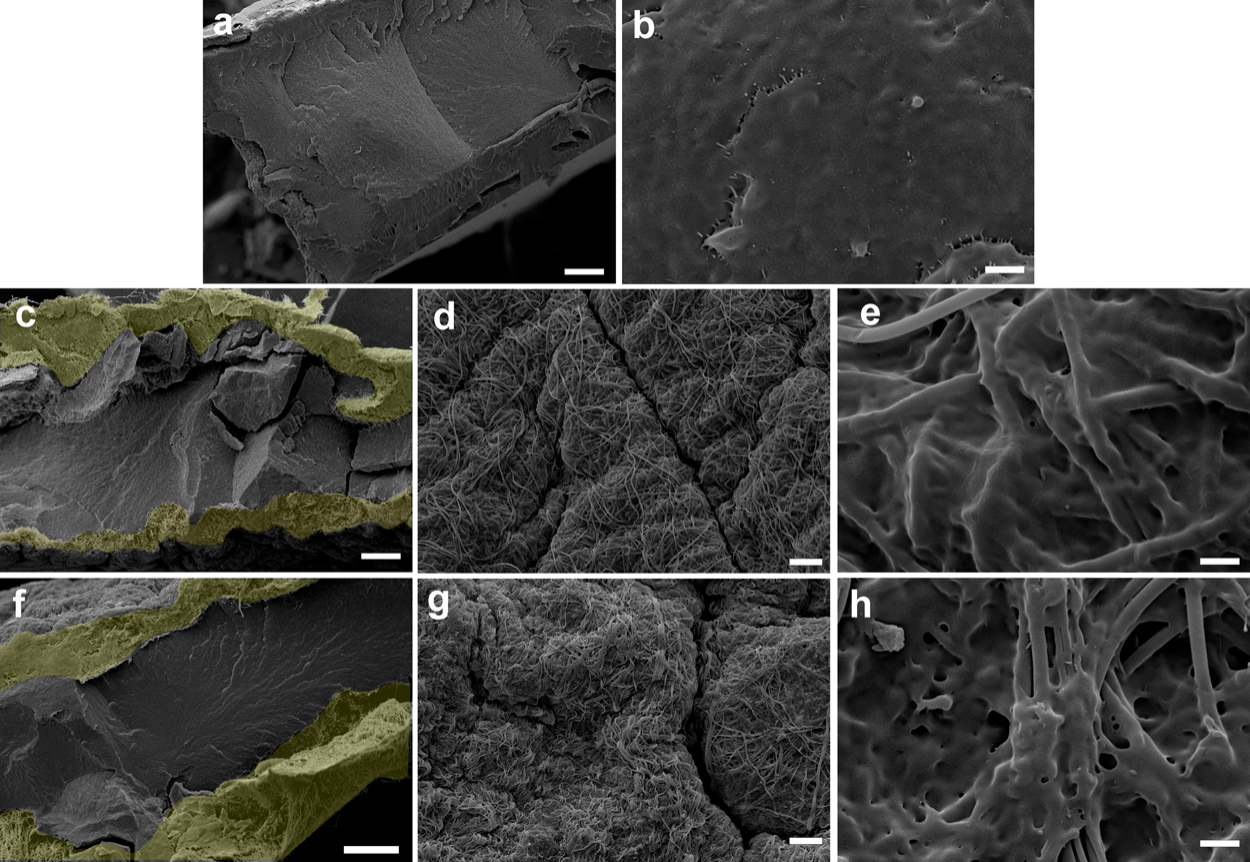
SEM micrographs of PHB–GVL gel (a,b), PA6,6/PHB–GVL composite (c–e), and PVA/PHB–GVL composite (f–h). (a,c,f) are cross-section images where the electrospun layers are highlighted in yellow; (b,d,e,g,h) are images of the external surfaces at different magnifications. Scale bar: 100 μm (a,c,f); 20 μm (d,g); 6 μm (b); 2 μm (e,h).

The dried PHB–GVL gel and the sandwich-like composites were observed by SEM both at the cross section and at the external surface (Figure 2). The PHB–GVL gel is a single layer structure (Figure 2a) with a relatively smooth surface (Figure 2b) devoid of any pores. In the cross-section images of the composites (Figure 2c,f), the two external electrospun layers (highlighted in yellow) are clearly distinguishable from the PHB–GVL core. It is pointed out that the thickness of the different layers in the swollen state is highly affected by the presence of the solvent and cannot be thus determined by SEM. However, SEM cross-sections highlight that the thickness of the external electrospun layers is significantly lower than that of the core, meaning that a few amount of fibers is enough to massively improve the mechanical properties, as discussed above (Figure 1h–m). Moreover, the shrinkage of the gel core, provoked by the solvent loss during the drying process, induced the curling of the external flexible electrospun layers, being well-adherent to the core part. Evidence of good adhesion is also provided by the images of composite external surfaces (Figure 2d,e,g,h). Here, the fibrous structure is clearly visible while the PHB–GVL component has well-penetrated and impregnated the pores. The presence of the gel component in the pores of the nonwoven, on one side, provides mechanical adhesion between the layers and, on the other side, is crucial for ensuring a good transfer of the cleaning solvent between the inner gel part of the composite and the external layer that will be directly in contact with the painting. Moreover, the microstructuration of the composite surface, thanks to the presence of the fibrous component, clearly determines an increase of surface roughness. From Figure 2e,h it is also evident that the gel component, besides filling the pores of the electrospun layer, also entirely surrounds and covers the surface of the fibers (see Figure S4 for additional images). This observation will be crucial for elucidating the cleaning mechanism of the composites.

**Figure 3:**
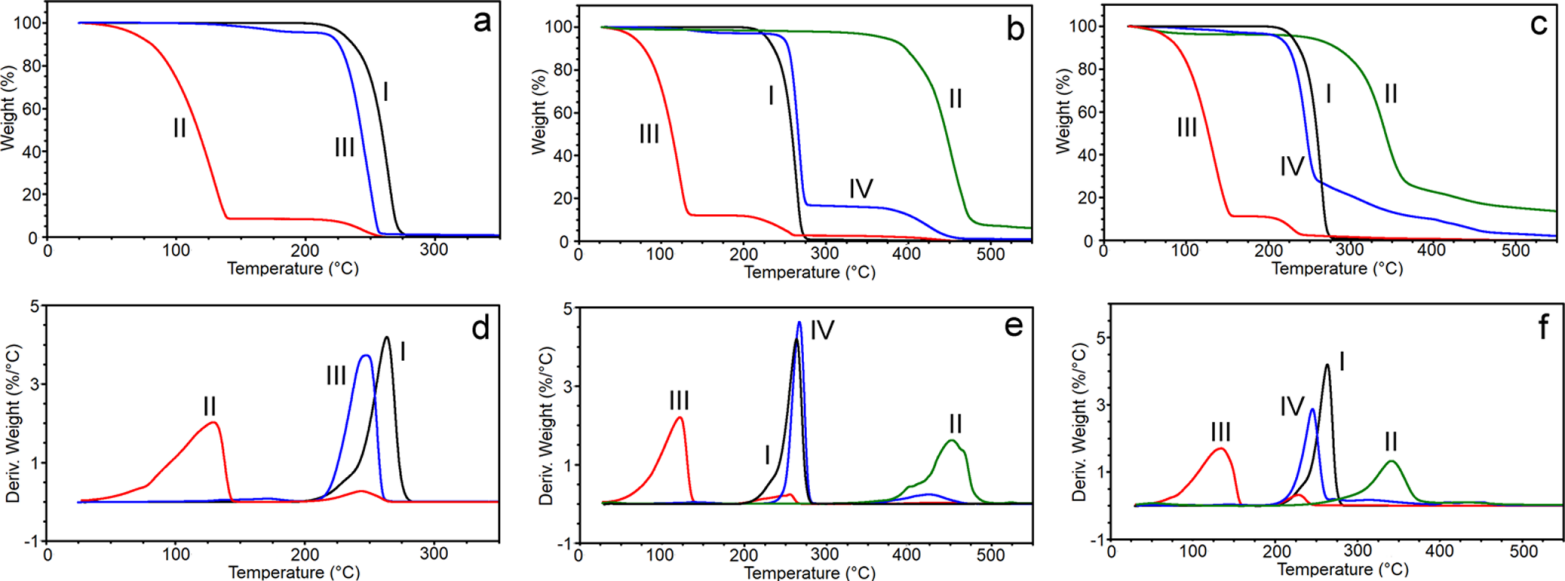
TGA (a–c) and corresponding derivative TGA (d–f) curves. In (a,d) (I) PHB, (II) PHB–GVL wet gel, and (III) PHB–GVL dried gel; in (b,e) (I) PHB, (II) PA6,6; (III) PA6,6/PHB–GVL wet composite, and (IV) PA6,6/PHB–GVL dried composite; in (c,f) (I) PHB, (II) PVA; (III) PVA/PHB–GVL wet composite, and (IV) PVA/PHB–GVL dried composite.

To gain information on the composition of the PHB–GVL gel and of the composites, TGA analysis was carried out. Figure 3 reports TGA and DTGA curves of both wet and dried gel and composites, together with the pure components (i.e., PHB, PA6,6, and PVA) for comparison. Corresponding weight losses in different temperature ranges are reported in Table 1. By considering the pure components, it is evident that PHB (curve I in Figure 3) completely degrades in the range 200–300 °C, PA6,6 (curve II in Figure 3b,e) almost completely degrades at temperatures higher than 300 °C, with a residual mass of 6 wt %, whereas PVA (curve II in Figure 3c,f) degrades in a multistep process from 200 to 550 °C and has a residual mass of 14 wt %. For both PVA and PA6,6, a small weight loss is recorded at low temperature ascribable to the evaporation of absorbed water because of the high hygroscopic nature of these polymers. The GVL solvent completely evaporated at temperature below 200 °C, as demonstrated by the comparison of the wet and dry gel. With these premises, it is possible to determine the composition of the different materials, both in terms of solvent content and polymer ratios in the composites. The PHB–GVL wet gel (curve II, Figure 3a,d) contains about 92 wt % of GVL (weight loss RT–200 °C), the remaining PHB weight fraction (8%) degrades in the range 200–300 °C, as expected. In the PA6,6/PHB–GVL system, the wet gel (curve III, Figure 3b,e) loses 88% of GVL weight before 200 °C and the remaining weight fraction is composed by PHB (9% weight loss 200–300 °C) and PA6,6 (3% weight loss 300–550 °C). Therefore, the polymer content in the PA6,6/PHB–GVL system is 12 wt %, which in turns contains 75 wt % of PHB and 25 wt % of PA6,6. The relative content of polymers is in line with the TGA analysis of the dried composite (curve IV, Figure 3b,e) that, albeit still displays a 3% of residual solvent, contains 80 wt % of PHB and 17 wt % of PA6,6. In the case of the PVA/PHB–GVL system, the wet composite (curve III, Figure 3c,f) is constituted by 89% of GVL and the remaining weight fraction is the polymer content (PHB + PVA). Here, the discrimination between the two types of polymers is difficult because of the PVA thermal degradation partially overlaps with that of PHB. However, in the dried composite (curve IV, Figure 3c,f) the weight loss in the range 200–300 °C is due to the concomitant degradation of PHB and of PVA, while the weight loss at 300–550 °C can be ascribable only to the degradation of PVA. By considering that pure PVA loses in this temperature range 71 wt % of its weight, it can be calculated that in the dried composite the content of PVA is about 27 wt %. Overall, TGA analysis suggests that both the gel and the composites contain about 90 wt % of solvent and that in the composites PHB content is in the range 73–80 wt % whereas the electrospun polymer amount is in the range 20–27 wt %, with the PVA/PHB–GVL composite being richer of fibers than the PA6,6/PHB–GVL one. This finding can be explained by considering that the PVA mats used for composite preparation were thicker than PA6,6 mats, as reported in Table S1.

**Table 1:** Thermogravimetric Data for Wet and Dried PHB–GVL Gel and Sandwich Composites, together with Data of Pure Components for Comparison

sample	Δ*m* % (RT–200 °C)	Δ*m* % (200–300 °C)	Δ*m* % (300–550 °C)	*m*_res_ %^a^
PHB	0	100	0	0
PA6,6	2	0	92	6
PVA	4	11	71	14
PHB–GVL wet	92	8	0	0
PHB–GVL dried	4	94	0	0
PA6,6/PHB–GVL wet	88	9	3	0
PA6,6/PHB–GVL dried	3	80	17	1
PVA/PHB–GVL wet	89	9	2	0
PVA/PHB–GVL dried	3	76	19	2

a Residual mass at 550 °C.

### Evaluation of the Cleaning Efficacy and Residuals

**Figure 4:**
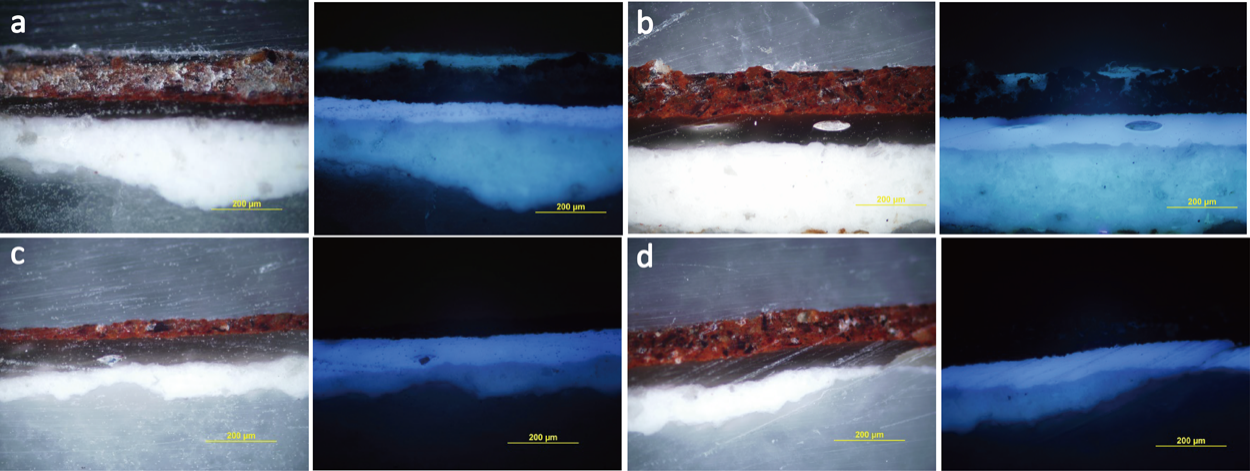
Cross-section microphotographs of mock-ups varnished with dammar, image under visible light (left) and image under UV illumination (right), (a) varnished area before cleaning, (b) after cleaning by PHB–GVL gel and cotton swab, (c) after cleaning by PVA/PHB–GVL gel and cotton swab, (d) after cleaning by PA6,6/PHB–GVL gel and cotton swab.

In order to investigate the cleaning performance of the different materials, mock-ups varnished with a 40 μm thick layer of dammar were used. Cross-sections of paint samples collected from the mock-ups before and after the cleaning treatment were observed with optical microscopy (Figure 4). It can be noted that, after cleaning with either PVA/PHB–GVL or PA6,6/PHB–GVL composites, the dammar varnish completely disappeared, whereas after the application of the PHB–GVL gel, some fluorescence spots, ascribable to residual dammar, were still visible on the painting surface. A higher cleaning efficacy was thus displayed by the sandwich composites compared to the gel, with no evident differences between the two type of composites.

**Figure 5:**
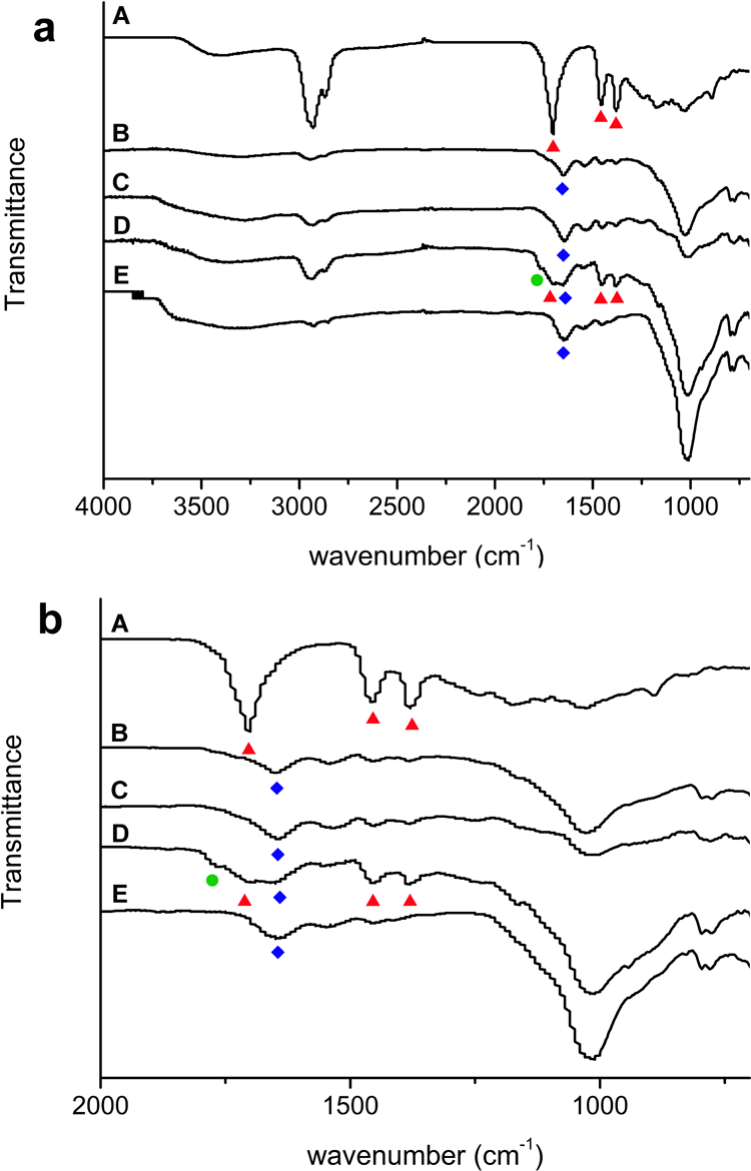
ATR-IR spectra acquired on the surface of the mock-ups: (A) dammar varnish area, (B) after cleaning with PVA/PHB–GVL composite, (C) after cleaning with PA6,6/PHB–GVL composite, (D) after cleaning with PHB–GVL gel, and (E) unvarnished area. The symbol ▲ indicates dammar characteristic bands, the symbol ⧫ indicates the characteristic bands of glue used as binder in the painting layer above the varnish, and the symbol ● indicates the GVL characteristic bands. (a) Spectra in the whole investigated wavelength range and (b) in the range 2000–675 cm^–1^.

The high cleaning performance of the composites was confirmed by FTIR microscopy in the ATR mode (Figure 5) performed on the mock-ups surface after 24 h from a 5 min treatment. FTIR spectra of dammar before cleaning and of the unvarnished substrate are also displayed for comparison. After cleaning with PHB–GVL gel, the characteristic bands of dammar (red triangles) at 1704 cm^–1^ (C=O stretching) and at 1455 and 1380 cm^–1^ (C–H_2_ scissoring) were just reduced. Moreover, residues of GVL were also observed as the band at 1767 cm^–1^ (green circle, C=O stretching) suggests. After the cleaning with the sandwich-like composites, the dammar C=O stretching band at 1704 cm^–1^ completely disappeared and no residue of GVL was observed. In addition, the glue binder characteristic bands (amide I at around 1650 cm^–1^ and amide II at around 1540 cm^–1^, blue squares), observed in the unvarnished paint layer, were also detected after cleaning with the composites, thus demonstrating the high efficiency of these new materials in removing the dammar coating, without leaving solvent residues. It is pointed out that no significant differences in the cleaning performance of PVA- and PA6,6-based composites were detected.

FTIR analyses in the ATR mode underline also that GVL residues were not detected after the application of the sandwich-like composites. To better quantify the solvent residues left on the painting after cleaning with the different materials HS-SPME was applied. This aspect is of key importance on a practical point of view because residual solvent on the paint surface could lead to a series of adverse effects. The evaluation of the amount of solvent released to the paint layer was aimed at comparing the different cleaning systems, thus acquiring information about their capability to retain the solvent. To make a realistic comparison, because 5 min treatment with PHB–GVL gel was not effective in completely removing the varnish, a second application of 5 min was also carried out for this material, after which the removal of the dammar was complete. In the case of sandwich-like composites, instead, only one 5 min-treatment was enough to achieve the same result in terms of cleaning efficiency. The amount of residual GVL detected after 2 and 24 h from the treatments are reported in Table 2. In the case of sandwich-like composites, PA6,6/PHB–GVL cleaned area showed a slightly higher amount of solvent than PVA/PHB–GVL cleaned area after 2 h from treatment, whereas, after 24 h, both cleaned areas contained a very small amount of residual solvent. Remarkably, in the PHB–GVL gel cleaned area a significantly higher amount of GVL remained when compared to the cleaning with the sandwich composites. The lower amount of solvent found in the case of composites can be the consequence of a slower release of GVL by these types of materials. This finding can be explained considering that, in the case of PHB–GVL gel, the solvent diffuses from the gel to the paint through the entire surface in contact with the paint, whereas, in the case of sandwich composites, a fraction of the surface in contact with the paint is constituted by fibers. The latter prevent the diffusion of the solvent toward the paint, albeit being surrounded by a thin layer of gel that, however, quickly solidify, thus interrupting the diffusion of GVL. In this way the action of the composite materials is more superficial and controlled with respect to the normal gel. Thus, by keeping constant the time of application, the amount of solvent transferred to the paint by the sandwich-like composites is less than that released by the gel. This reduced amount of solvent is, however, enough to swell and soften the dammar that can be easily removed from the paint surface leaving behind no residues, contrary to what observed by the PHB–GVL gel (Figure 4). It is worth mentioning that the different solvent content measured after 2 h in the areas cleaned by the two composites can be ascribable to the different electrospun layer thickness. Indeed, albeit PVA and PA6,6 fibers have similar morphologies in terms of fiber diameter, the PVA layer is slightly thicker than PA6,6 one, thus providing a higher solvent retention ability.^44−46^

**Table 2:** Amount of GVL Residues (wt %) after 2 and 24 h from the Cleaning Treatments with Gels and Cotton Swab

sample	time of treatment (min)	GVL content at 2 h (wt %)	GVL content at 24 h (wt %)
PHB–GVL	5	0.449 ± 0.120	0.273 ± 0.127
PHB–GVL	10	0.543 ± 0.060	0.338 ± 0.001
PA6,6/PHB–GVL	5	0.271 ± 0.029	0.170 ± 0.018
PVA/PHB–GVL	5	0.160 ± 0.001	0.143 ± 0.021

**Figure 6:**
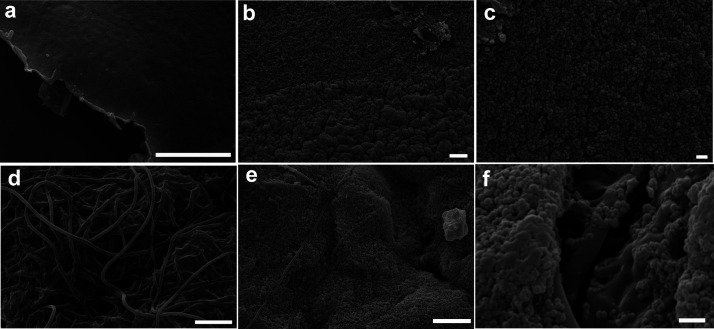
Representative SEM images of: (a) PHB–GVL surface before cleaning; (b,c) PHB–GVL surface after cleaning external; (d) PA6,6/PHB–GVL composite surface before cleaning; (e,f) PA6,6/PHB–GVL composite surface after cleaning. Scale bar: 10 μm (a,b,d,e); 1 μm (c,f). PVA/PHB–GVL composite surface after cleaning displays the same morphology of PA6,6/PHB–GVL.

**Figure 7:**
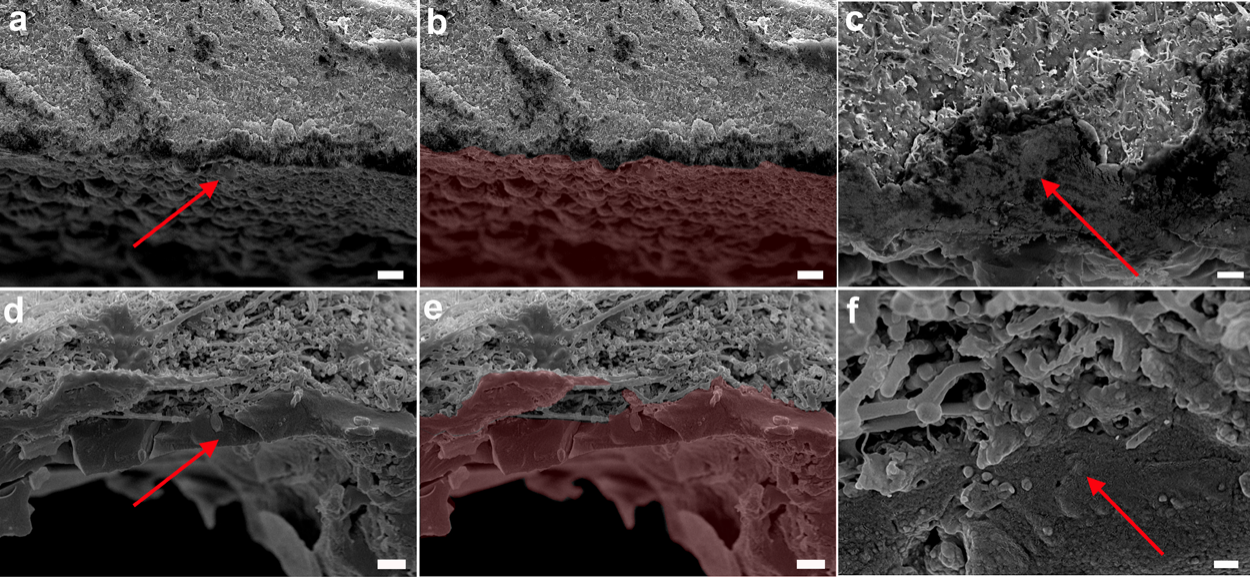
SEM images of material cross-sections after cleaning: (a–c) PHB–GVL gel and (d–f) PA6,6/PHB–GVL composite. Red arrows indicate the side of the section covered by the dammar. In (b,e) the dammar layer attached to the composite has been colored in red. Scale bar: 10 μm (a,b,d,e); 2 μm (c,f). PVA/PHB–GVL composite cross-section after cleaning displays the same morphology of PA6,6/PHB–GVL.

To understand why the sandwich-like composites show higher cleaning efficiency, SEM observation of the samples after cleaning was performed (Figures 6 and 7). Figure 6 reports SEM images of the gel and composite surface before (a,d) and after (b,c,e,f) cleaning. Both in the case of gel and of composite, the side that came in contact with the varnish is covered by a continuous layer of dammar, that appears as agglomerates of small spherical nanoparticles. In the composites, dammar particles appear to be well in contact with the fibres covered by the gel (Figure 6f). Additional representative SEM images can be found in Figure S5.

Figure 7 shows the cross-sections of organogel (a–c) and composite (d–f) after cleaning. Here, it is possible to observe that the dammar layer is well anchored to the fibrous structure, having partially penetrated the pores of the non-woven (see Figure 7d–f). Additional representative SEM images can be found in Figure S5. Differently, the dammar layer collected by the PHB–GVL gel appears not well attached to the material (Figure 7a–c).

**Figure 8:**
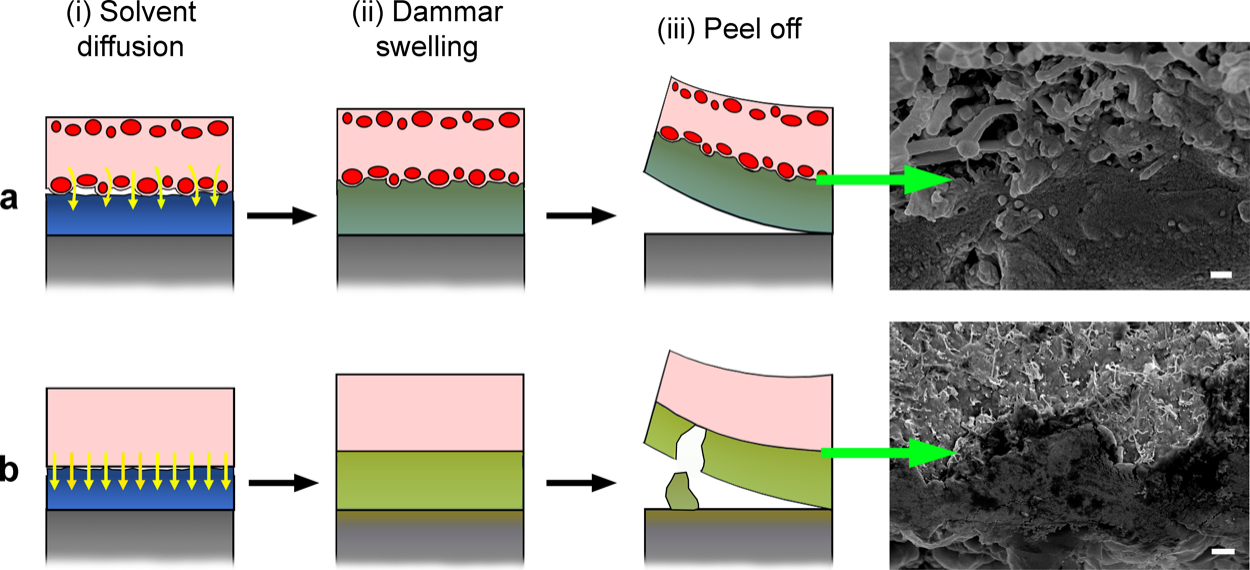
Schematic representation of the mode of action of (a) sandwich-like composites and (b) PHB–GVL organogel for the cleaning of dammar varnish. (i) Solvent diffusion from the gel phase to the varnish (solvent is depicted by yellow arrows): in the sandwich-like composites GVL diffusion is limited by the presence of the fibrous layer (fibre sections are depicted as red circles). (ii) dammar swelling: dammar swells after GVL absorption from the gel phase by following either the rough surface morphology of the composites (a) or the smooth surface of the organogel (b). Higher swelling is expected in the case of organogel compared to the composites, given the higher GVL diffusion from the former. (iii) Removal of the cleaning material by peeling off: dammar layer is effectively peeled off by using composites thanks to its good mechanical adhesion with the rough surface (see SEM image), whereas residual solvent and fragments of dammar can remain on the paint when organogel is used, due to its worse adhesion with the gel (see SEM image).

Figure 8 shows a schematic representation of the possible mode of action of sandwich-like composites compared to pure organogel. The cleaning procedure can be considered as a three-step process: (i) solvent diffusion from the gel phase to the varnish; (ii) dammar swelling and (iii) removal of the cleaning material. In the first step the GVL quickly diffuses from the pure organogel into the dammar layer whereas in the composites the solvent release is slowed down by the presence of the fibers, as previously speculated to explain HS-SPME results. GVL is thus absorbed by the dammar that, as a consequence, swells. The soft dammar can penetrate the pore of the electrospun layer to a certain extent and can thus be easily removed from the paint when the composites are peeled off. Therefore, the micro-structured rough superficial morphology and the interconnected porosity in the sandwich composites provide a higher surface which enables the entrapment of dammar within the porosity and the establishment of a high mechanical adhesion between the varnish layer and the surface composite. This permits to achieve a higher cleaning performance when compared to the single layer gel. The latter, instead, possessing a smoother surface, displays a lower attitude to completely remove the dammar varnish from the substrate and, at the same time, is more susceptible to cohesive failure, with the risk that gel residues may remain on the object after cleaning. This mechanism is supported by the results obtained also with other techniques which, as previously discussed, suggest that the chemical nature of the fibrous component has no evident effect on the cleaning performances. Since the surface of the fibers is completely covered by the PHB component (Figure S4), it turns out that the dammar and the fibers do not directly come in contact during cleaning and the same chemical interactions are established between dammar and composites and dammar and pure gel. Therefore, the role of the fibers is only to provide structural support for the gel and to increase the surface roughness, with the consequent increase of mechanical adhesion between PHB and dammar.

## Conclusions

This study reports the first attempt to combine electrospun nonwoven fabrics with organogels for cleaning purposes. New materials with a sandwich-like structure, consisting of two external layers of electrospun fibers and a core of organogel, have been developed and tested for painting cleaning. The presence of the external electrospun layers confers to the composite new properties and advanced functionalities. First, the electrospun nonwoven exerts the function of mechanically supporting component, thus massively improving the mechanical resistance of the system, as demonstrated by mechanical tests. This aspect is of key importance because facilitates the use and the handling of the cleaning material, especially in the laying and peeling-off procedures, reducing the presence of residual debris on the paints. Cleaning tests and SEM observations highlighted that the presence of a microstructured surface with an interconnected porosity provided by the electrospun layer ensures, on one side, the possibility for the active solvent to diffuse from the inner gel toward the paint surface with a more superficial action than the normal gel and, on the other side, act as anchoring sites for the swollen dammar. Overall, the new developed cleaning systems may be better handled, have better cleaning performances, and are safer toward the cultural object than the neat gel, thanks to their controlled and superficial action. Efforts are currently devoted to further investigate the role played by electrospun materials in cleaning procedures. Thus, new tests, based on the application of PVA and PA with the use of different neat green solvents, are being carried out. Given these exceptional results, such a kind of materials may find application for the cleaning and disinfection of other delicate surfaces, in personal care, and hygiene fields.

## Supporting Information Available

Composite geometry; differential scanning calorimetry; rheological properties of PHB–GVL solution; and SEM characterization (PDF)
