# Supplementary material for: Organogel Coupled with Microstructured Electrospun Polymeric Nonwovens for the Effective Cleaning of Sensitive Surfaces
Source: ACS Appl Mater Interfaces. 2020 Aug 21;12(35):39620–9. doi: 10.1021/acsami.0c09543 (PMC8009474; doi:10.1021/acsami.0c09543)
Supplement: Supplementary file 1 — am0c09543_si_001.pdf [file am0c09543_si_001.pdf]

Supporting Information

Organogel coupled with microstructured electrospun polymeric nonwovens for the effective cleaning of sensitive surfaces

Yiming Jia[1,2], Giorgia Sciutto[1], Rocco Mazzeo[1], Chiara Samorì[3], Maria Letizia Focarete[4,5], Silvia Prati[1], Chiara Gualandi[4,6]**

[1] Department of Chemistry "G. Ciamician", Microchemistry and Microscopy Art Diagnostic Laboratory (M2ADL), University of Bologna, Via Guaccimanni 42, 48121 Ravenna, Italy

[2] Chongqing Cultural Heritage Research Institute, 400013 Chongqing, China

[3] Department of Chemistry "G. Ciamician", University of Bologna, Via Sant'Alberto 163, 48123 Ravenna, Italy

[4] Department of Chemistry "Giacomo Ciamician" and INSTM UdR of Bologna, University of Bologna, Via Selmi 2, 40126 Bologna, Italy

[5] Health Sciences & Technologies (HST) CIRI, University of Bologna, Via Tolara di Sopra 41/E, 40064 Ozzano Emilia Bologna, Italy

[6] Interdepartmental Center for Industrial Research on Advanced Applications in Mechanical Engineering and Materials Technology, CIRI-MAM, University of Bologna, Viale Risorgimento, 2, 40136 Bologna, Italy

Corresponding Authors: Dr. Chiara Gualandi: c.gualandi@unibo.it; Prof. Silvia Prati: s.prati@unibo.it.

1. Composite geometry

Table S1. Thickness of the mat layers used to prepare the composites and of the corresponding final composites.

sample	Bottom mat thickness (μm)	Upper mat thickness (μm)	Composite thickness (mm)
PVA/PHB-GVL	93	112	1.74
PVA/PHB-GVL	91	86	1.68
PVA/PHB-GVL	98	92	1.86
PA6,6/PHB-GVL	78	75	1.83
PA6,6/PHB-GVL	83	74	1.80
PA6,6/PHB-GVL	76	75	1.78

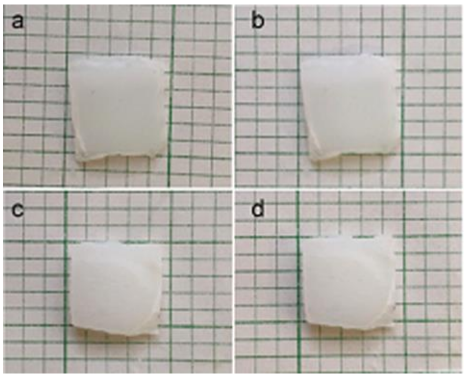

Figure S1. Pictures of (a) as-prepared PVA/PHB-GVL composite, (b) PVA/PHB-GVL composite after 30 min at room temperature, (c) as-prepared PA6,6/PHB-GVL composite and (d) PA6,6/PHB-GVL composite after 30 min at room temperature.

2. Differential Scanning Calorimetry

Figure S3 reports the first DSC heating scans of electrospun mats of PVA (Figure S3a) and PA 6,6 (Figure S3b) before (black curves) and after contact with GVL at 110 °C and drying (blue curves). Electrospun PVA is semicrystalline, with a glass transition temperature (T_g) around 66 °C and a melting temperature (T_m) at 193 °C with a melting endotherm (ΔH_m) of 46 J/g (Figure S3a, black). Electrospun PA6,6 is also semicrystalline, with a T_g around 70 °C and a T_m at 260 °C with a ΔH_m of 75 J/g (Figure S3b, black). After pouring a drop of GVL at 110°C on the mats, to simulate the step of PHB-GVL dropping during composite preparation, the DSC curve of the samples did not change (Figure S3, blue), meaning that the structural properties of both fibers are not affected by the contact with GVL.

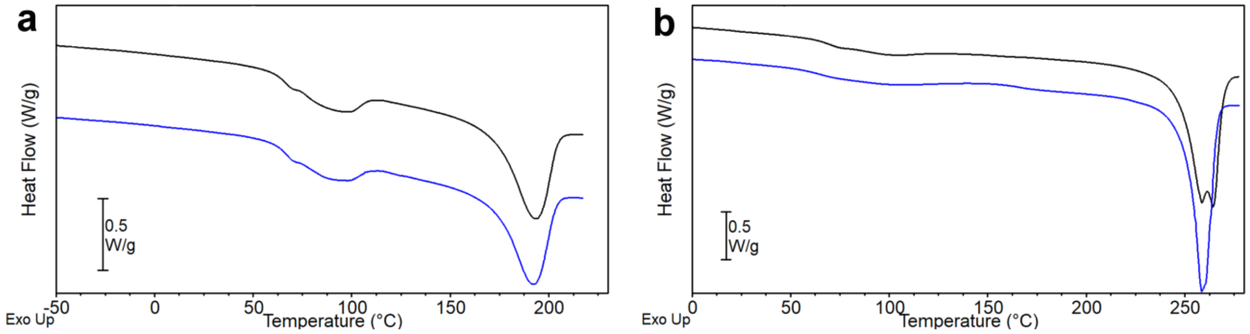

Figure S2. First DSC heating scans of PVA (a) and PA6,6 (b) electrospun fibers. Black curves correspond to the virgin fibers and the blue curves to the same samples after contact with GVL at 110°C.

3. Rheological properties of PHB-GVL solution

PHB-GVL mixture is a homogeneous solution when kept at high temperature but it undergoes a liquid-to-solid transition upon cooling. The gelation process leads to a jelly-like state with an enormous increase in the viscosity and storage modulus. Figure S1 reports the temperature sweep experiments performed on the PHB-GVL solution at different cooling rates. It is verified that the gelation temperature (T_{gel}), taken at the crossover of the storage modulus (G') and loss modulus (G''), is in the range 75-113 °C, and increases with the decrease of cooling rate. The dependence of T_{gel} on the cooling rate is typical of physical gels since the sol-gel transition is a kinetically controlled process. Figure S1d reports the viscosity as a function of temperature during a cooling

scan at 10 °C/min. the viscosity of the PHB-GVL solution is constant up to about 80 °C when gelification occurs.

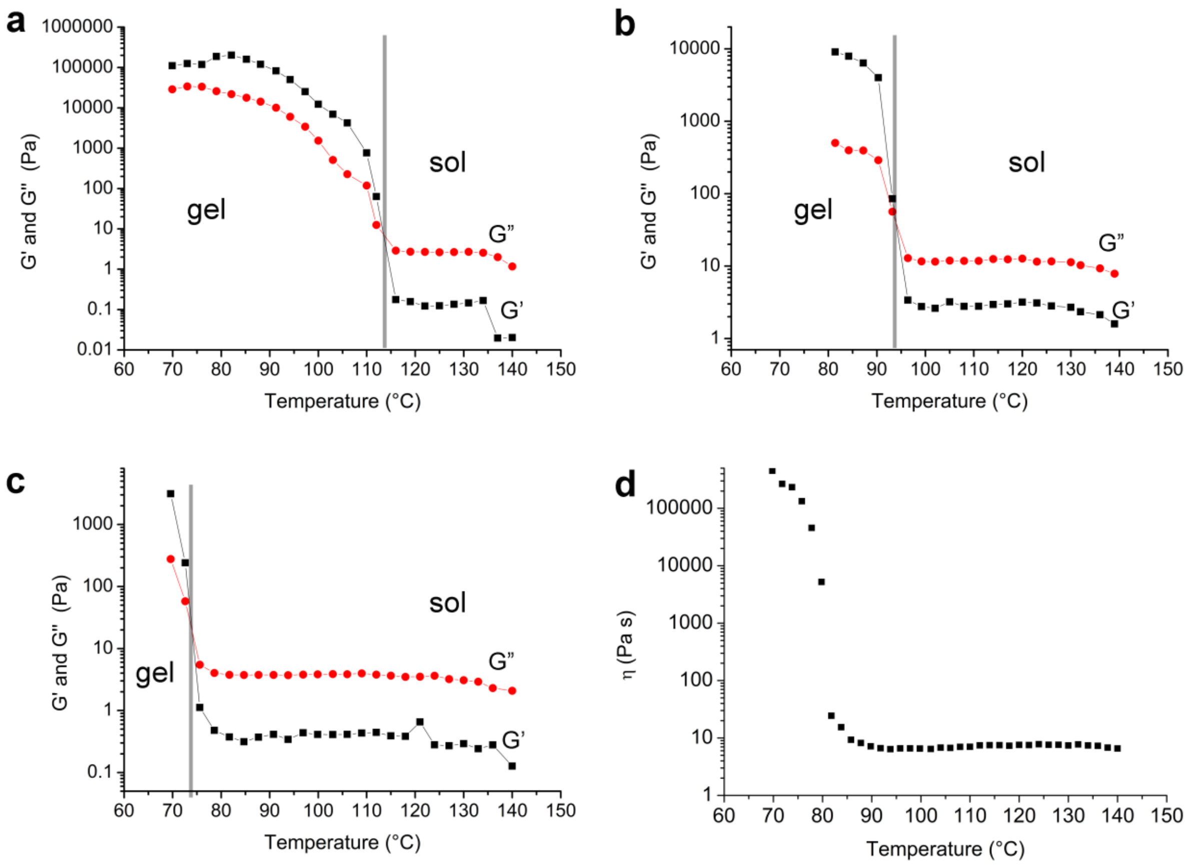

Figure S3. Temperature sweep experiments performed on PHB-GVL solution at different cooling rates: (a) 1 °C/min, (b) 5 °C/min and (c) 10 °C/min. d) change of viscosity during a cooling scan at 10 °C/min.

4. SEM characterization

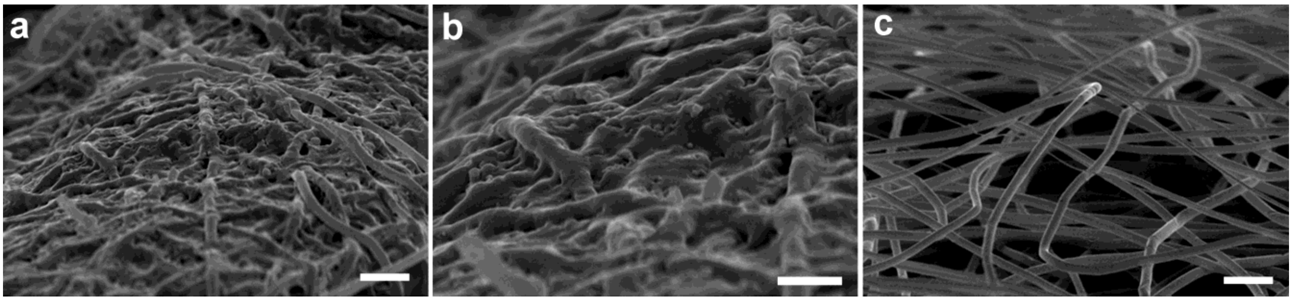

Figure S4. Representative SEM images of fibers embedded in the PHB-GVL gel when assembled in the composite (a and b) and of original electrospun fibers (c). Scale bar: 5 µm (a and c); 3 µm

(b). All samples were observed with an angle of about 60° with respect to the electron source. Images clearly show that in the composites the gel component fills the pores and completely covers the surface of the fibers.

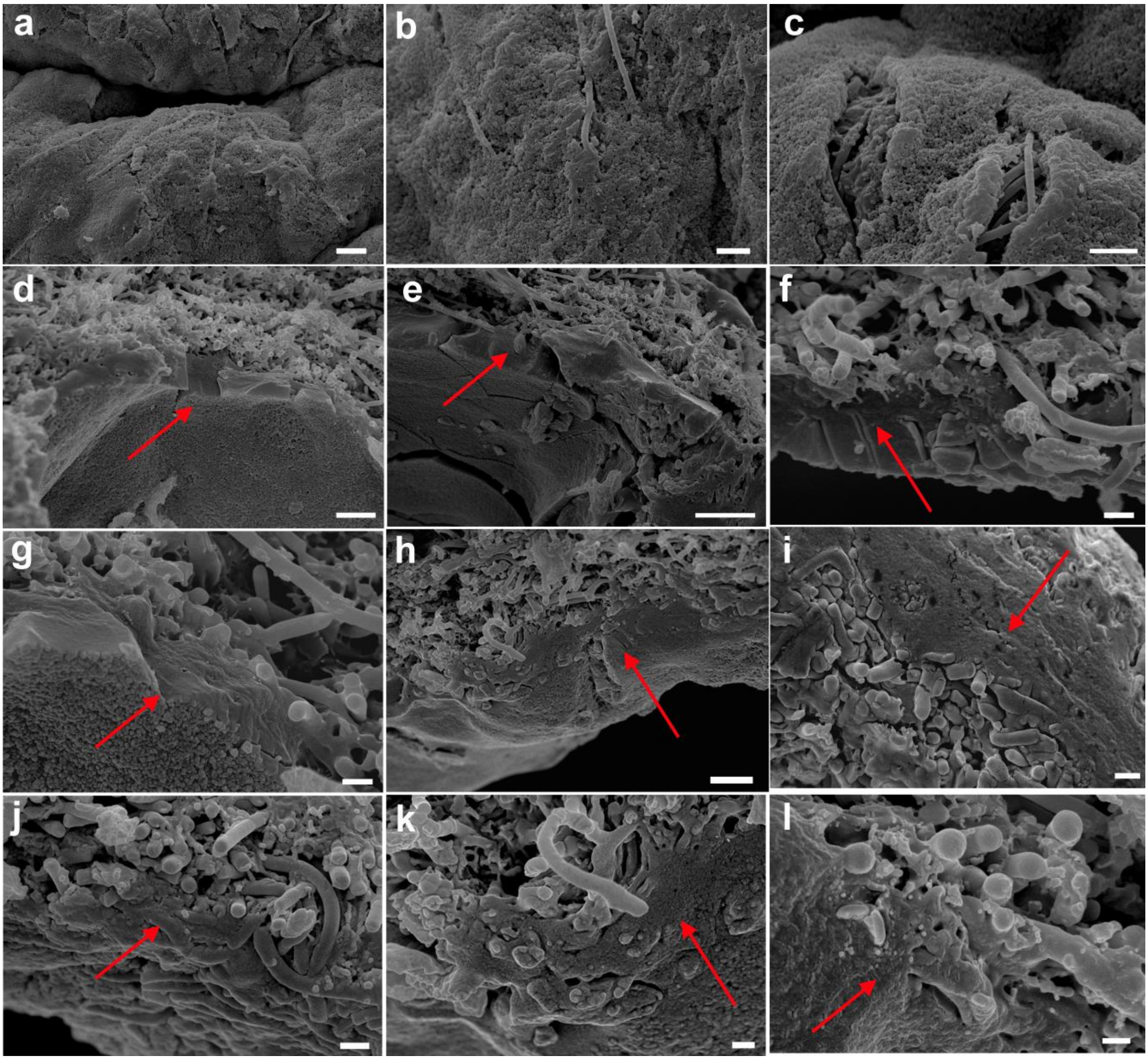

Figure S5. Representative SEM images of the composites after cleaning. The side of the composite in contact with the mock-up has been observed both at the surface (a, b, and c) and in cross-section (d-l). Red arrows indicate the side of the section covered by the dammar. Scale bar: 10 µm (a, d, e); 5 µm (b); 4 µm (c); 2 µm (f, g, h, i, j, k); 1 µm (l).
